# Identification of RSK substrates using an analog-sensitive kinase approach

**DOI:** 10.1016/j.jbc.2024.105739

**Published:** 2024-02-10

**Authors:** Belén Lizcano-Perret, Didier Vertommen, Gaëtan Herinckx, Viviane Calabrese, Laurent Gatto, Philippe P. Roux, Thomas Michiels

**Affiliations:** 1 Molecular Virology Unit, de Duve Institute, Université Catholique de Louvain, Brussels, Belgium; 2 MASSPROT Platform, de Duve Institute, Université Catholique de Louvain, Brussels, Belgium; 3 Institute for Research in Immunology and Cancer (IRIC), Université de Montréal, Montreal, Quebec, Canada; 4 Computational Biology and Bioinformatics Unit, de Duve Institute, Université Catholique de Louvain, Brussels, Belgium; 5 Faculty of Medicine, Department of Pathology and Cell Biology, Université de Montréal, Montreal, Quebec, Canada

**Keywords:** RSK1, RSK4, MAPK pathway, kinase, phosphorylation, substrate, TRIM33, analog-sensitive kinase

## Abstract

The p90 ribosomal S6 kinases (RSK) family of serine/threonine kinases comprises four isoforms (RSK1-4) that lie downstream of the ERK1/2 mitogen-activated protein kinase pathway. RSKs are implicated in fine tuning of cellular processes such as translation, transcription, proliferation, and motility. Previous work showed that pathogens such as Cardioviruses could hijack any of the four RSK isoforms to inhibit PKR activation or to disrupt cellular nucleocytoplasmic trafficking. In contrast, some reports suggest nonredundant functions for distinct RSK isoforms, whereas Coffin-Lowry syndrome has only been associated with mutations in the gene encoding RSK2. In this work, we used the analog-sensitive kinase strategy to ask whether the cellular substrates of distinct RSK isoforms differ. We compared the substrates of two of the most distant RSK isoforms: RSK1 and RSK4. We identified a series of potential substrates for both RSKs in cells and validated RanBP3, PDCD4, IRS2, and ZC3H11A as substrates of both RSK1 and RSK4, and SORBS2 as an RSK1 substrate. In addition, using mutagenesis and inhibitors, we confirmed analog-sensitive kinase data showing that endogenous RSKs phosphorylate TRIM33 at S1119. Our data thus identify a series of potential RSK substrates and suggest that the substrates of RSK1 and RSK4 largely overlap and that the specificity of the various RSK isoforms likely depends on their cell- or tissue-specific expression pattern.

p90 Ribosomal protein S6 kinases (RSK) are Ser/Thr protein kinases activated by extracellular signal-regulated kinases 1/2 (ERK1/2) in the ERK–mitogen-activated protein kinase (MAPK) pathway. The ERK–MAPK pathway is a key cellular pathway linked to cellular processes such as cell proliferation, survival, and motility (1, 2, 3, 4). It is therefore not surprising that this pathway has been tightly connected to cancer with more than 30% of cancer mutations occurring in proteins within this pathway (5). Activation of the ERK–MAPK pathway by different factors such as growth factors, hormones, and chemokines induces the autophosphorylation of a tyrosine kinase receptor (*e.g.*, EGFR, FGFR), which, through an activation cascade, induces Ras activation. Ras then activates Raf which activates MEK1/2 which in turn activates ERK1/2 to finally phosphorylate and activate RSKs downstream of the pathway (1, 2, 3, 4).

Mammalian cells contain four very closely related (73–80% amino acid identity) isoforms of RSK: RSK1 (*RPS6KA1*), RSK2 (*RPS6KA3*), RSK3 (*RPS6KA2*), and RSK4 (*RPS6KA6*) that are encoded by distinct genes. RSKs are particular protein kinases as they possess two kinase domains belonging to different kinase families (6, 7). It is thought that RSKs evolved from the fusion of two kinases. The N-terminal kinase domain (NTKD) belongs to the AGC (protein kinase A, G and C) family and the C-terminal kinase domain (CTKD) to the CaMK (Ca^2+^/calmodulin-dependent protein kinase) family. The main reported function of the CTKD is to activate the NTKD which then phosphorylates cellular substrates. RSKs’ canonical activation starts by the phosphorylation of the CTKD by ERK1/2. This phosphorylation activates the CTKD which then phosphorylates the linker region contained between the NTKD and the CTKD (8). The phosphorylation in this linker region creates a docking site for 3-phosphoinositide-dependent protein kinase 1 which binds to this region and in turn phosphorylates the NTKD, thus activating RSKs (9, 10). Interestingly, the RSK4 isoform was reported to be constitutively active, and its activation to be 3-phosphoinositide-dependent protein kinase 1-independent (11). The consensus phosphorylation motif of RSKs is similar to that of other members of the AGC family R/K-x-R-x-x-S∗/T∗ (∗ indicates phosphorylation) and is thought to be shared by the four RSK isoforms (12). The region in the active site of RSK NTKD, which accommodates substrate residues, is very well conserved in all isoforms (91% identity). This indicates that these kinases likely phosphorylate very similar motifs.

RNA-seq data of the human protein atlas (proteinatlas.org) (13) indicate that all RSK isoforms are ubiquitously expressed but that their expression level varies in a tissue-specific fashion. RSK4 expression level is low in most organs. The RSK1 isoform is most strongly expressed in the gastrointestinal tract, bone marrow, and lymphoid tissues; RSK2 is mainly found in endocrine organs as the parathyroid gland, muscle tissue, and the liver and RSK3 is mainly found in the brain, endocrine tissue, heart, and lung. In the mouse, during embryogenesis, RSK expression also differs depending on the isoform. Expression of RSK4 seems to be low throughout development (14). RSK2 is also expressed at a low level until late embryonic stages, where its expression levels up in some tissues. Finally, RSK1 and RSK3 expression is high in some tissues during development and varies according to the embryogenesis stage (15).

Even though the four RSK isoforms have a high amino acid sequence identity, only mutations in RSK2 have been linked to the Coffin-Lowry syndrome, a disease characterized by growth deficits and mental retardation (16). This suggests that the different RSK isoforms have different functions and therefore possibly different substrates. An alternative explanation is that RSK2 would be expressed more or less than other isoforms in specific cell types linked to disease onset. For RSK1-2, which are the most studied RSK isoforms, common substrates have been identified (17). In a previous work, we observed that pathogens such as cardioviruses could equally hijack any of the four RSK isoforms to disrupt nucleocytoplasmic trafficking in the cell (18) or to inhibit the antiviral kinase interferon-induced double-stranded RNA–activated kinase PKR (19). In line with these data, a recent study showed that all four RSK isoforms were phosphorylating very similar peptides in a peptide array type of screening (12). Other works however support nonredundant activities of the distinct RSK isoforms in cells, likely linked to differences in the range of substrates phosphorylated by the different kinase isoforms (20).

Therefore, despite many substrates were identified for the various RSK isoforms, whether they exhibit some substrate specificity is still an open question. In this work, we addressed this question by focusing on two of the four RSK isoforms: RSK1 and RSK4. RSK1 is one of the most expressed and studied with more than 70 substrates identified in phosphosite.org, and RSK4 is the most different of all the RSK isoforms and the less studied with only three substrates identified in phosphosite.org. We used the analog-sensitive kinase strategy to identify RSK1 and RSK4 substrates. This method originally developed by the group of Kevan Shokat (21, 22) allows the identification, in living permeabilized cells, of kinase substrates. It is complementary to other proximity-based or inhibitor-based approaches (23, 24, 25), which have been used in the quest of RSK substrates and has the advantage to identify substrates that are direct targets of the kinase of interest.

## Results

### Constructing the analog-sensitive RSK1 and RSK4

**Figure 1:**
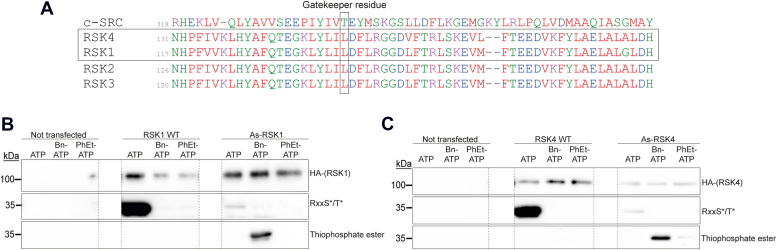
**Producing and testing the analog-sensitive RSKs.***A*, alignment of c-SRC kinase domain with the NTKD of RSKs. Sequences were taken from uniprot (c-SRC: P00523, RSK1: Q15418, RSK2: P51812, RSK3: Q15349, RSK4: Q9UK32). The gatekeeper residue aligns with Thr338 of c-SRC and is supposedly localized after two hydrophobic amino acids and followed by an acidic amino acid and a hydrophobic amino acid. *B* and *C*, As-RSK1 (*B*) and As-RSK4 (*C*) thiophosphorylate substrates *in vitro.* HEK293T cells were transfected with plasmids coding for RSKs-WT or As-RSKs. Twenty four hours post transfection, RSKs were immunoprecipitated and used in an *in vitro* kinase assay in the presence of a recombinant substrate (GST-S6) and ATP or A∗TP-S (N6-Bn-ATP-γ-S or N6-PhEt-ATP-γ-S). Thiophosphorylated substrates were then alkylated by the addition of p-nitrobenzylmesylate (PNBM) to form a thiophosphate ester that can be specifically recognized with the anti-thiophosphate ester antibody. Western blots show the presence of RSK (anti-HA), the phosphorylation of the GST-S6 substrate (anti-RxxS∗/T∗ - antibody recognizing RSK phosphorylated substrates), and the thiophosphorylation of the substrate (anti-thiophosphate ester). *Dashed lines* between lanes indicate deletion of irrelevant lanes from the same membrane. As, analog-sensitive; RSK, ribosomal S6 kinase.

To identify specific substrates of RSK1 and RSK4, the isoforms displaying the most differences, we used the analog-sensitive kinase system (21). This system is based on mutations enlarging the ATP-binding pocket of a kinase of interest (RSK1 or RSK4 in our case) so that it can accommodate a bulkier ATP analog that cannot be used by other kinases in the cell. For substrate identification, we used the bulkier ATP-analog: N6-alkylated ATP-γ-S (A∗TP-S), which contains a thiophosphate as the gamma phosphate. Therefore, unlike substrates of other kinases, substrates of the analog-sensitive kinase become thiophosphorylated. The gatekeeper residues, which restrict the kinase ATP pocket size, were identified as Leu141 for RSK1 and Leu152 for RSK4, based on the rule originally derived from c-SRC (26) and on the alignment with RSK2, which was recently successfully converted to analog-sensitive (18) (Fig. 1*A*). Leu141 (RSK1) and Leu152 (RSK4) were mutated into the smaller residue Ala. *In vitro* kinase assays confirmed that the mutated kinases retained their catalytic activity (Fig. 1, *B* and *C*, anti-RxxS∗/T∗ antibody – ATP lane). Two different A∗TP-S analogs, N6-Bn-ATP-γ-S or N6-PhEt-ATP-γ-S, were tested in the assays. In order to detect thiophosphorylated substrates, an alkylation reaction with p-nitrobenzylmesylate (PNBM) was made after the *in vitro* kinase assay, which allows the conversion of the thiophosphate into a thiophosphate ester that can be specifically recognized with an anti-thiophosphate ester antibody. Both analog-sensitive(As)-RSKs were able to thiophosphorylate the substrate (Fig. 1, *B* and *C*, anti-thiophosphate ester) especially with the N6-Bn-ATP-γ-S analog. This analog was thus chosen for all the following experiments.

### Identification of RSK1 and RSK4 substrates in HeLa cells

**Figure 2:**
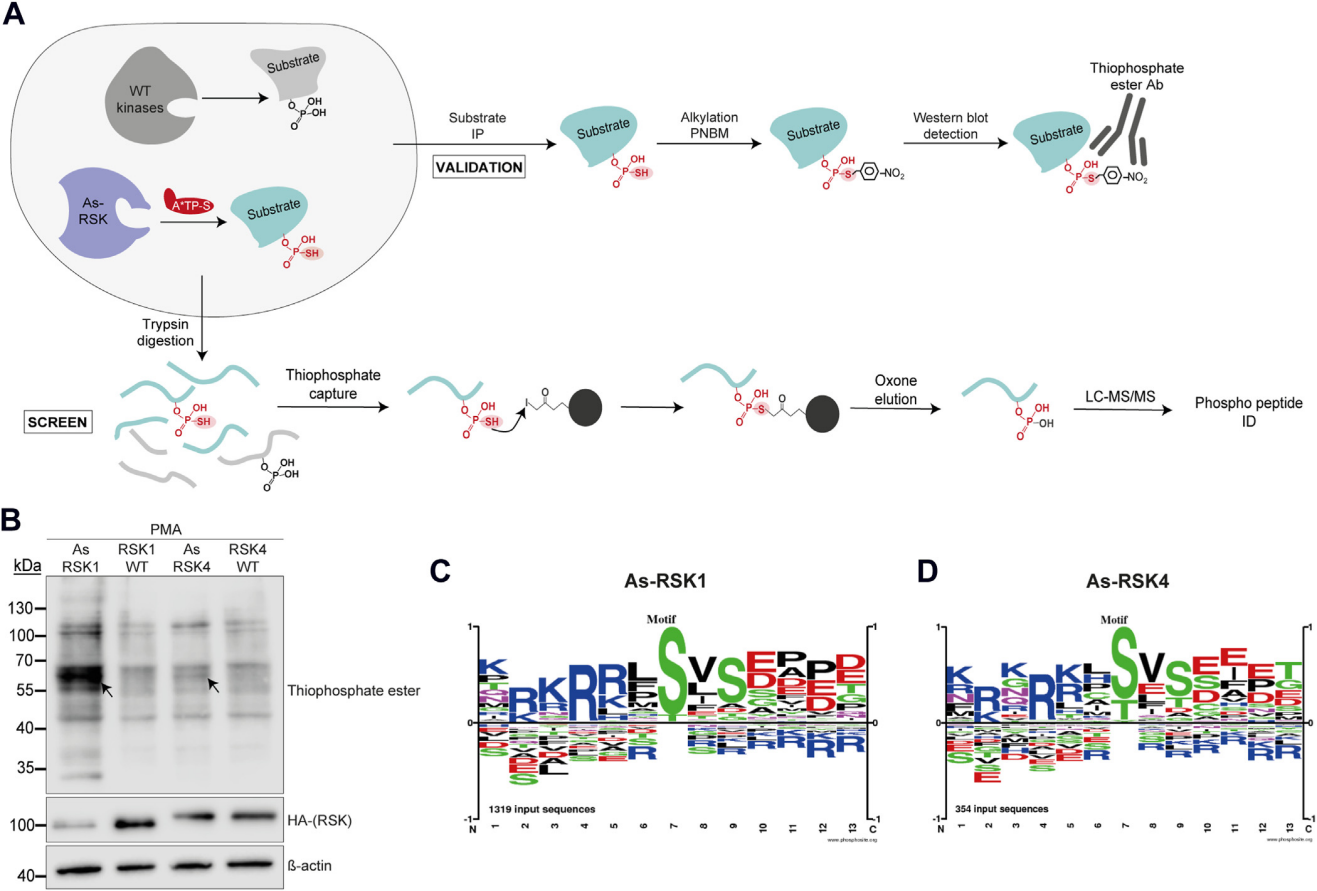
**As-RSK1 and As-RSK4 thiophosphorylated peptides enrichment.***A*, cartoon showing the layout of the enrichment experiment (screen) and the immunoprecipitation experiment (validation). The As-RSK thiophosphorylate specific substrates in cells. Screen: after cell lysis, proteins are digested with trypsin and thiophosphorylated peptides are captured with an iodoacetyl resin. After multiple washes, peptides are eluted with oxone. Phosphorylated peptides are then introduced in the LC-MS/MS and identified. Validation: after cell lysis, a candidate substrate is immunoprecipitated. Immunoprecipitated proteins are then alkylated with PNBM. In Western blot, the thiophosphate esterylated protein will be specifically recognized with the anti-thiophosphate ester antibody. *B*, As-RSKs thiophosphorylate substrates after PMA activation. Western blot showing levels of expression of the (HA)-RSKs and thiophosphorylated substrates. *Arrows* point to a band, which is stronger in the analog-sensitive condition (specific thiophosphorylation). ß-actin was detected as a loading control. *C* and *D*, phosphorylation motif derived from phosphorylated peptides identified after thiophospho-enrichment (n = 3). Motif was derived, using the phosphosite.org tool, from phospho-peptides that were more abundant in As-RSK than in RSK-WT conditions. RSK, ribosomal S6 kinase; As, analog-sensitive; PMA, phorbol 12-myristate 13-acetate; PNBM, p-nitrobenzylmesylate.

The WT forms of RSK1 and RSK4 (RSK1-WT and RSK4-WT) and their analog-sensitive counterparts (As-RSK1 and As-RSK4) were individually and stably expressed by transduction in HeLa RSK-TKO cells. RSK-TKO cells had their genes coding for RSK1, 2, and 3 inactivated by CRISPR-Cas9 and fail to express RSK4 (19). The following cells were obtained: HeLa RSK1-WT, HeLa As-RSK1, HeLa RSK4-WT, and HeLa As-RSK4, where the only RSK isoform expressed is the transduced one. Attempts at identifying thiophosphate-esterylated proteins by mass spectrometry (MS) in these cells were unsuccessful. The reason behind these unsuccessful experiments may be that the thiophosphate ester moiety is modified or cleaved during the LC-MS/MS process. We therefore decided to use another method based on the enrichment of thiophosphorylated peptides developed by Schaffer *et al.* (22) to identify RSK substrates (Fig. 2*A*). With this method, the thiophosphorylated peptides are enriched through binding of their thiol group to an iodoacetyl resin and then eluted as phospho-peptides, which can be readily detected by LC-MS/MS (Fig. 2*A*-screen). It is noteworthy that cysteine-containing peptides can also be retained on the iodoacetyl column, but these peptides should be detected equally with WT- and As-kinases. As a validation method for the identification of RSK1 and RSK4 substrates, the candidate proteins can be immunoprecipitated and, after the alkylation reaction with PNBM, their thiophosphate ester can be detected by Western blot with the specific antibody (Fig. 2*A*-validation).

To identify RSK1 and RSK4 substrates in HeLa cells, cells re-expressing each of the constructs, RSK1-WT, As-RSK1, RSK4-WT, or As-RSK4, were treated with phorbol 12-myristate 13-acetate (PMA) for 15 min to activate the MAPK pathway. Then, cells were permeabilized to allow the entry of the A∗TP-S analog and the thiophosphorylation reaction to happen. Cells were then lysed, proteins digested, and thiophosphorylated peptides captured. After elution with oxone, phosphorylated peptides were identified by MS (n = 3) (Pride repository dataset identifier PXD047020 and 10.6019/PXD047020). As a control of the efficacy of the thiophosphorylation reaction in the cells, part of the lysate was alkylated with PNBM, and proteins were detected by Western blot with the anti-thiophosphate ester antibody. Figure 2*B* shows that thiophosphorylation readily occurred as the signal was more intense for As-RSKs than for RSKs-WT. RSK1 activity was however stronger than RSK4 activity as there were many more thiophosphorylated proteins detected for As-RSK1. Thiophosphorylation of proteins by As-RSK4 was more modest, yet distinguishable from background (Fig. 2*B*).

We derived phosphorylation motifs from the phosphorylated peptides enriched in As-RSK1 *versus* RSK1-WT (Figs. 2*C* and S1) and As-RSK4 *versus* RSK4-WT (Figs. 2*D* and S1). To this end, around 1300 (As-RSK1) and 350 (As-RSK4) phosphorylated peptides were aligned. These motifs show that RSK1 and RSK4 have very similar consensus phosphorylation sequences, which also match the known consensus for RSKs: R/K-x-R-x-x-S∗/T∗. As expected, the phosphorylation motifs derived from phospho-peptides detected in cells expressing WT RSKs (RSK1-WT or RSK4-WT) do not show a specific consensus sequence, as these peptides were likely mostly cysteine-containing peptides that were phosphorylated on Ser, Thr, or Tyr by any kinase in the cell (Fig. S1). The difference between the number of detected peptides phosphorylated by As-RSK1 (1300) and As-RSK4 (350) indicates that As-RSK1 is more active than As-RSK4 or/and that the two kinases are not activated similarly by the PMA treatment.

As a first analysis of the MS data, phospho-peptides that were present in at least two of the three replicates in As-RSK conditions were kept for further analysis. These phospho-peptides were sorted by calculating a score based on the ratio between the number of peptide spectrum matches (PSMs) detected for As-RSK and for RSK-WT. Thus, candidates that have high amount of PSMs in As-RSKs and low in RSK-WT will have a higher score. Table 1 shows the 50 best-ranked phospho-peptides detected for As-RSK4 and their corresponding scores for As-RSK1. Interestingly, 49 out of the top 50 peptides identified for As-RSK4 were also found in at least two of the three As-RSK1 screens. These data strongly suggest that, in our experimental conditions (HeLa cells), these two kinases share many common substrates.

**Table 1:** The 50 best-ranked As-RSK4 substrate candidates and their corresponding scores for RSK1

**Figure 3:**
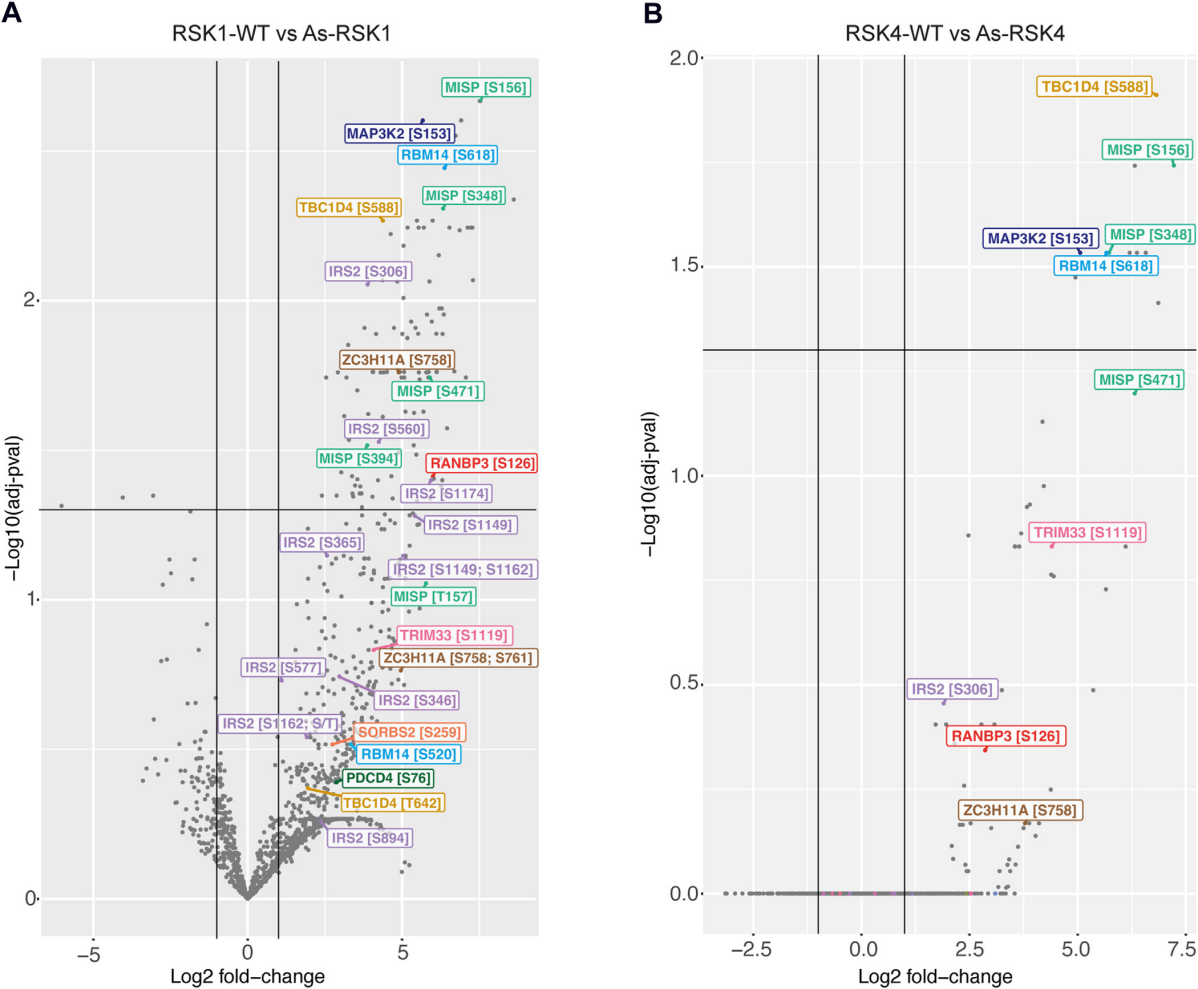
**Identification of peptides thiophosphorylated by As-RSK1 or As-RSK4.** Volcano plots showing comparisons between phospho-peptides (*A*) in RSK1-WT *versus* As-RSK1 or (*B*) RSK4-WT *versus* As-RSK4. Peptides localized in the *top right* square have an adjusted *p*-value <0.05 and Log2 fold change >1. As, analog-sensitive.

A ProDA (Probabilistic Dropout Analysis) statistical analysis of the data was then performed on pairwise comparisons between As-RSK1 *versus* RSK1-WT and As-RSK4 *versus* RSK4-WT data (27). Volcano plots are shown in Figure 3. Not surprisingly, there are less statistically significant peptides identified for As-RSK4 than for As-RSK1, in line with the lower thiophosphorylation activity of RSK4 detected by Western blot. Many previously identified RSK1 substrates were detected for both As-RSK1 and As-RSK4, including Ran-binding protein 3 (RanBP3) (28), TBC1 domain family member 4 (TBC1D4) (29), and programmed cell death protein 4 (PDCD4) (23) (Fig. 3), supporting the effectiveness of the As-kinase strategy.

### IRS2, ZC3H11A, and SORBS2 are substrates of RSK4 and/or RSK1

**Figure 4:**
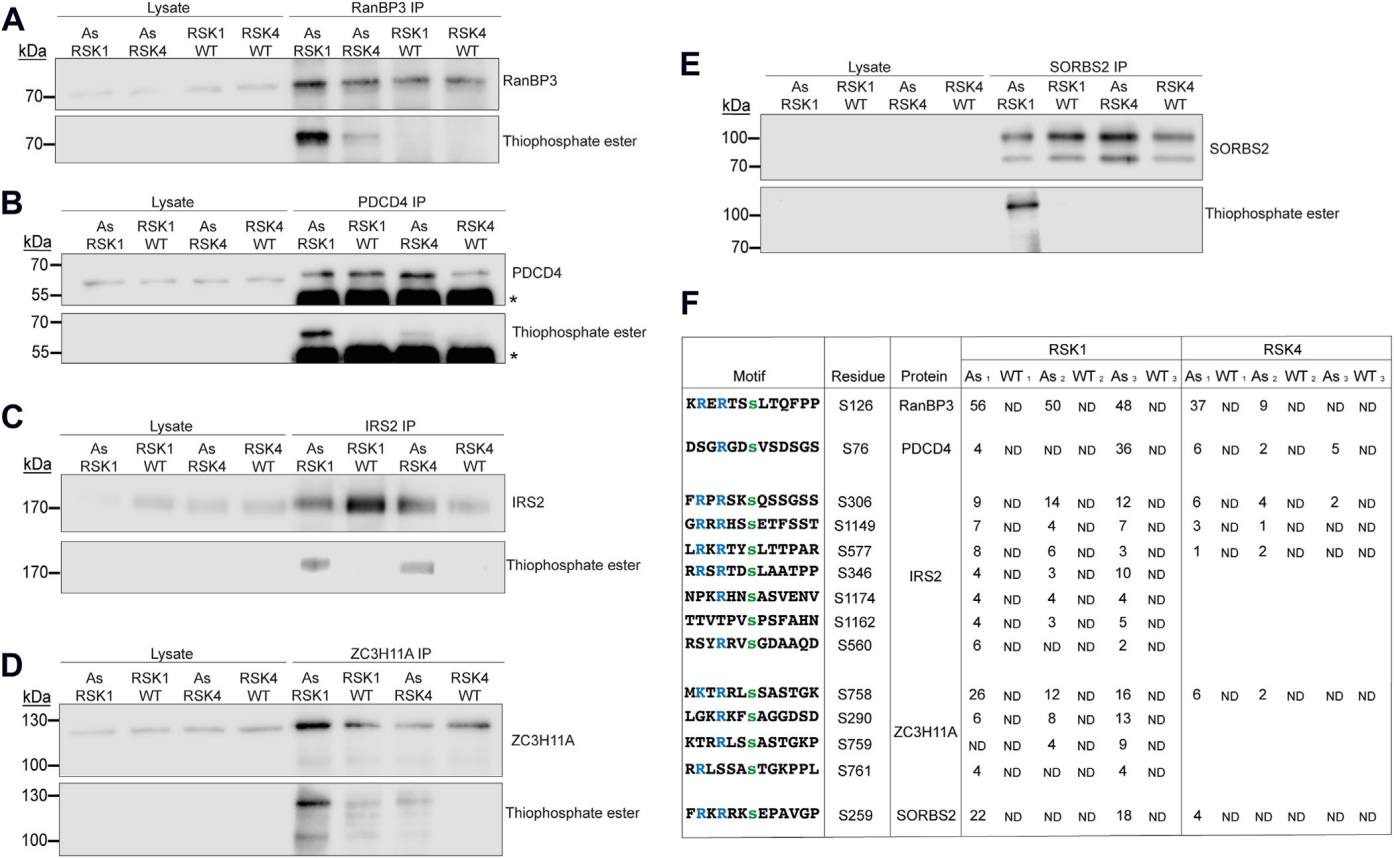
**IRS2, ZC3H11A, and SORBS2 are substrates of RSK1/4.***A* and *B*, Western blots of RSK known substrates (*A*) RanBP3 and (*B*) PDCD4 immunoprecipitation and its thiophosphorylation. *C*–*E*, Western blots of RSK substrate candidates (*C*) IRS2, (*D*) ZC3H11A, and (*E*) SORBS2 immunoprecipitation and its thiophosphorylation. *F*, table showing the PSMs for each candidate’s phospho-peptide identified in the thiophosphorylated peptides enrichment experiment (n = 3). ND = non detected. Blots sample order is different between panels. ∗: antibody heavy chain. IRS2, insulin receptor substrate 2; PDCD4, programmed cell death protein 4; PSM, peptide spectrum match; RanBP3, Ran-binding protein 3; RSK, ribosomal S6 kinase.

To validate our screening results, selected known and “new” (non-listed as substrates in phosphositeplus.org) RSK1 or RSK4 substrates were immunoprecipitated from cells following the treatment with the ATP analog, and their thiophosphorylation status was tested by Western blot after alkylation (Fig. 2*A*-validation). Known RSK1 substrates tested included RanBP3 (adj. *p*-val <0.05 in RSK1 screen) and PDCD4 (adj. *p*-val >0.05 in RSK1 screen). For other candidates, we selected proteins whose phospho-peptides were detected in at least two replicates with As-RSKs and undetected in all three replicates with WT RSKs (Table 2). The selected candidates were insulin receptor substrate 2 (IRS2), Sorbin and SH3 domain-containing protein 2 (SORBS2), and Zinc finger CCCH domain-containing protein 11A (ZC3H11A). Both IRS2 and ZC3H11A had low adj. *p*-values in the RSK1 screen (Fig. 3*A*). SORBS2, however, had an adj. *p*-val >0.05 but was still selected since its *p*-value was lower than the one of PDCD4, which is a known RSK substrate. All selected proteins were successfully immunoprecipitated from cells after PMA treatment, and their thiophosphorylation status was tested by Western blot. Positive controls, RanBP3 and PDCD4, were readily thiophosphorylated by both As-RSK1 and As-RSK4 (Fig. 4, *A* and *B*). Interestingly, a single (thio)-phosphorylated residue was detected for these two proteins, which matches the residue known to be phosphorylated by RSK (Fig. 4*F*). In line with the data shown in Figure 2*B*, these proteins were less thiophosphorylated by As-RSK4 than by As-RSK1.

**Table 2:** The 50 best-ranked As-RSK1 substrate candidates

We then tested three candidate RSK substrates: IRS2, ZC3H11A, and SORBS2. IRS2 belongs to the IRS family which is composed of three isoforms in humans (IRS1, IRS2, and IRS4) (30). These proteins are implicated in mitogenesis, glucose metabolism, and are important intermediates of the insulin signaling pathway since they bind to insulin receptors. Their effect on mitogenesis and metabolism is mediated through activation of the Ras/MAPK and PI3K pathways (31, 32, 33, 34). Insulin has been shown to activate RSK1 in L6 myocytes, inducing RSK1-mediated phosphorylation of IRS1 S1101. This phosphorylation regulates glucose metabolism since inhibition of RSK prevented insulin resistance (35). Our data show that IRS2, which is the main IRS isoform expressed in HeLa cells, according to the proteinatlas.org, is thiophosphorylated by both As-RSK1 and As-RSK4 (Fig. 4*C*). In the thiophospho-peptide enrichment experiment, we identified seven different IRS2 phosphorylation sites for As-RSK1 and three for As-RSK4 (Fig. 4*F*). One of these is S1149 which is the equivalent of IRS1 S1101 that was previously found to be phosphorylated by RSK1 (35). Our data suggest that RSKs play an important role in insulin signaling since not only S1149/S1101 but several other residues are phosphorylated by RSKs in IRS2 (Fig. 4*F*).

ZC3H11A was recently identified as being part of the TREX complex, which is implicated in mRNA biogenesis and mRNA nuclear export (36, 37, 38). Interestingly, ZC3H11A overexpression correlates with the occurrence of KRAS mutations in lung adenocarcinoma (39), providing a link between the Ras/MAPK pathway and ZC3H11A. Our data show that both As-RSK1 and As-RSK4 thiophosphorylate ZC3H11A (Fig. 4*D*). Although some background signal was detected, the signal was clearly more intense for As-kinases than for WT-kinases for both RSK1 and RSK4. Multiple ZC3H11A phosphorylation sites were identified in our MS screen, including one that contains a clear consensus sequence of RSK phosphorylation and others that contain a closely related sequence (Fig. 4*F*).

SORBS2, also called Arg kinase-binding protein 2 (ArgBP2), is a protein belonging to the SoHo (sorbin homology) family of adapter proteins (40). There are multiple splicing isoforms of SORBS2 ranging from 70 to 140 kDa. Two isoforms of ∼70 kDa and ∼100 kDa were detected in our cells (Fig. 4*E*). SORBS2 is implicated in cellular motility and contains SH3 domains that mediate the interaction with regulators of the actin cytoskeleton. Recent data indicate that SORBS2 might be a tumor suppressor since its expression is decreased during pancreatic cancer transformation and its overexpression in metastatic cell lines inhibits cellular migration (41, 42). Our results indicate that the ∼100 kDa isoform of SORBS2 is thiophosphorylated by As-RSK1 (Fig. 4*E*). In the case of As-RSK4, we did not detect any thiophosphorylation, which could either mean that RSK4 does not phosphorylate SORBS2 or that the phospho-peptide was less abundant and fell under the detection level. Only one phosphorylated residue (S259) was detected once for As-RSK4 in our screen (Fig. 4*F*). This residue (S259) has previously been reported to be phosphorylated by PKA to reduce the interaction of SORBS2 with α-actinin (43). Our results might implicate a role for RSKs in SORBS2-regulated cell motility.

These results indicate that our analog-sensitive RSK screen is reliable since known and new RSK substrates were identified with this approach. We therefore compiled in Table 2 the 50 best-ranked candidate substrates of RSK1 according to PSM-based scores calculated as for Table 1.

### TRIM33 is phosphorylated by endogenous RSK at S1119

To further test the reliability of the analog-sensitive RSK screen data, we selected another candidate substrate to test its phosphorylation by endogenous RSK using a more conventional approach based on mutagenesis and kinase inhibitors. The E3 ubiquitin-protein ligase TRIM33 was chosen since only one phosphorylated residue (S1119) of this protein had been detected in our screen. In addition, the TRIM33 phospho-peptide was well-ranked (present in the top 50 candidates of both RSK1 and RSK4) (Tables 1 and 2) and had a low (though not significant) adj. *p*-value (Table 2 and Fig. 3*A*).

TRIM33, also called transcriptional intermediary factor 1γ (TIF1γ), is a ubiquitin ligase that can also act as a transcription cofactor (44). TRIM33 has been implicated in the inhibition of TGF-ß signaling by acting at two different levels. It ubiquitinates SMAD4, which blocks its interaction with the other SMADs (45) and binds phospho-SMAD2/3, thereby competing with SMAD4 (46). Thus, in both cases, TRIM33 blocks the TGF-ß/SMAD pathway. Link of TRIM33 to cancer has also been studied and was shown to either act as a tumor suppressor or as a tumor promoter, depending on the cell type examined (44).

**Figure 5:**
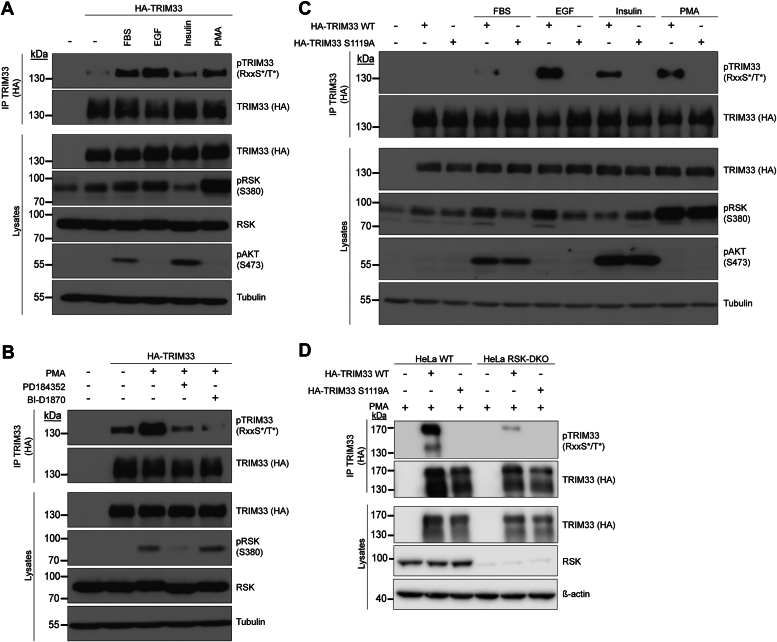
**TRIM33 is phosphorylated by RSK at S1119.***A*, HEK293 cells were transfected with HA-tagged TRIM33, serum-starved overnight, and stimulated for 10 or 20 min with fetal bovine serum (FBS; 10%), EGF (25 ng/ml), insulin (100 nM), or PMA (50 ng/ml). Immunoprecipitated TRIM33 was then assayed for phosphorylation with a phospho-motif antibody that recognizes the RxxS∗/T∗ consensus motif. *B*, same as in (*A*), except cells were pretreated with PD184352 (10 μM) or BI-D1870 (10 μM) for 30 min prior to PMA stimulation. *C*, as in (*A*), except cells were transfected with HA-tagged TRIM33 WT or the S1119A unphosphorylable mutant. *D*, detection of phospho-RxxS∗/T∗ in TRIM33-WT and -S1119A in WT and RSK-DKO HeLa cells. Cells transfected with the HA-tagged TRIM33 constructs were stimulated for 30 min with PMA prior to HA immunoprecipitation. PMA, phorbol 12-myristate 13-acetate; RSK, ribosomal S6 kinase.

To test if TRIM33 is phosphorylated by RSK, HA-TRIM33 was immunoprecipitated from transfected HEK293 cells that were treated with different RSK activators (fetal bovine serum (FBS), EGF, insulin, and PMA). Then, TRIM33 phosphorylation status was assessed by using the anti-RSK phospho-substrate antibody (RxxS∗/T∗). As can be seen in Figure 5*A*, although RSK activation was only partial in some samples (insulin treatment), TRIM33 was readily phosphorylated in an RSK phospho-motif after treatment with all inducers. This phosphorylation was decreased after treatment with the MEK1/2 inhibitor PD184352 and with the RSK inhibitor BI-D1870, indicating its dependence on RSK (Fig. 5*B*). In our analog-sensitive screen, we identified S1119 as the (thio)-phosphorylated residue. We therefore expressed a non-phosphorylatable mutant form of TRIM33 where serine 1119 was mutated to alanine. The phosphorylation of TRIM33 in an RSK phospho-motif was lost after RSK activation when TRIM33 was mutated in S1119A, confirming that S1119 is phosphorylated by RSK (Fig. 5*C*). Since kinase inhibitors can have off-target effects, we assessed TRIM33 phosphorylation in HeLa RSK-KO cells transfected with the TRIM33-WT and -S1119A constructs. As can be seen in Figure 5*D* and Fig. S2, TRIM33 phosphorylation was strongly diminished in RSK-DKO and RSK-TKO cells compared to WT cells. The slight phosphorylation of TRIM33 seen in RSK-KO cells may be due to residual RSK activity of those cells (see Experimental procedures) or to the activity of another kinase, which phosphorylates the same residue. Taken together, these results indicate that endogenous RSKs phosphorylate TRIM33 at S1119, thus validating the data of the analog-sensitive RSK screen.

## Discussion

RSKs form a family of kinases activated by the ERK–MAPK pathway. These kinases are linked to many cellular processes such as translation and transcription regulation but also cellular proliferation, differentiation, and motility. Even though the four RSK isoforms are being more and more studied (mainly in the context of cancer), the question of their substrate and function redundancy is still open. In this work, we addressed this question by studying the two most different RSK isoforms: RSK1 and RSK4. Our data suggest that RSK1 and RSK4 share many substrates. On one hand, consensus sequences derived from As-RSK1 and As-RSK4 data are very similar (Fig. 2, *C* and *D*). On the other hand, 49 out of the 50 top RSK4 substrates identified in our screen also occurred in the list of RSK1 substrates. Also, four out of the five substrates validated by co-immunoprecipitation and thiophosphate detection, RanBP3, PDCD4, IRS2, and ZC3H11A, were phosphorylated by both RSK1 and RSK4. In our experiments, As-RSK4 was less active than As-RSK1 at phosphorylating substrates (Figs. 2, *B*–*D* and 4, *A*–*E*). Thus, lack of detection of an RSK1 substrate as an RSK4 substrate might be due to the sensitivity of the technique. Taken together, this indicates that the two RSK isoforms share many substrates. This is in accordance with the work of Johnson, Yaron *et. al.* (12), in which they identified *in vitro* the phosphorylation consensus of more than 300 kinases. In their work, all RSKs clustered together when kinases were organized based on their amino acid motif selectivity, indicating that these kinases phosphorylate substrates that are either the same or contain very similar phosphorylation sites.

Our data confirm the identity of previously reported RSK substrates and identify “new” substrates. It is noteworthy that a series of substrates identified in this work, using the As-kinase strategy, also occur in the data of other RSKs proteomics or phospho-proteomics analyses, where more traditional technologies (including BioID and inhibitors) were used to identify RSK interactors and potential substrates (23, 24, 25).

A Gene Ontology search identified “non-membrane-bounded organelle assembly,” “mitotic cell cycle process,” and “cytoskeleton organization” as enriched pathways for the substrates identified in Tables 1 and 2. In addition, some of the identified substrates contribute to other pathways such as translation regulation (EIF2A, EIF4B), insulin signaling (IRS2), or cytokine signaling (TRIM33). Thus, our data confirm that RSKs have pleiotropic activities in the cell.

A strength of the As-kinase approach is that it not only identifies substrates but also the phosphorylated residue. In the case of TRIM33, both As-RSK1 and As-RSK4 screens identified S1119 as the phosphorylated residue. Using a more “traditional” approach based on mutagenesis, kinase inhibitors, and RSK-KO cells, we confirmed that RSKs phosphorylate TRIM33 specifically at S1119. This reinforces our As-kinase screen results. Thus, an asset of the As-kinase system is the fact that it detects direct RSK substrates in cells with high specificity.

Many works have addressed the influence of RSKs in different cancers. Overall, the data suggest that RSK1 and RSK2 are pro-cancerous and RSK3 and RSK4 are more tumor-suppressive. The data however point to more subtle differences according to the cancer type. Expression or activation of RSK1-2 have been linked to different types of cancer including prostate cancer (47), leukemia (48), breast cancer (49, 50), and lung cancer (51). However, also in lung cancer, RSK1 has been shown to be a tumor suppressor since its depletion increased cell motility and enhanced metastatic potential, whereas RSK4 depletion had the opposite impact and was acting as a pro-cancerous agent (52). This is contrary to other observations where RSK3 and RSK4 expression has been shown to be lower in some cancers including breast (53, 54), colorectal (55), and ovarian cancer (56). Interestingly, a recent work by the group of J.W. Ramos showed that even in the same cell-type, two RSKs regulate different transcription programs. By using microarray analysis in glioblastoma-derived cells were RSK1 or RSK2 were knocked-down, this group showed that these two kinases do not induce the same gene transcription patterns (20). In brief, RSKs and their link to cancer clearly depends on the RSK isoform and the type of cancer.

In conclusion, the As-kinase strategy is a powerful tool, which enables the identification of kinase substrates in the cell, at the phosphorylated residue level. The use of this technique allowed to identify a series of RSK1 and RSK4 substrates. Although our work does not rule out some extent of substrate specificity, it suggests that the substrate ranges of different RSK isoform largely overlap and that the differences observed between physiological activities of the four RSK isoforms, such as RSK2-specific mutations in the Coffin-Lowry syndrome (16), may be more related to the different expression levels of these isoforms in different cell types.

## Experimental procedures

### Cells

HeLa cells used in this study are the HeLa-M subclone (kindly provided by R.H. SIlvermann). HeLa RSK-DKO (RSK1-2) and RSK-TKO (RSK1-2-3) cells were previously obtained using the CRISPR-Cas9 technology (19). These cells were checked by RNA sequencing. HeLa-M cells express very little RSK3 and almost no RSK4 mRNA. Thus, DKO and TKO cell lines are virtually full RSK-KO. One transcribed allele is however detected carrying a 81 nt in-frame deletion in RSK1 (19).

HeLa RSK1-WT, As-RSK1, RSK4-WT, and As-RSK4 cells are HeLa RSK-TKO cells transduced with the lentiviral vectors TM1116, BLP32, TM1119, and BLP34, respectively. The transduced populations of cells were selected with 2 mg/ml of G418 (Roche). Expression of RSKs WT or As-RSKs was checked by Western blot. HEK293T cells used in this work were a kind gift of F. Tangy (Pasteur Institute, Paris). HeLa and HEK293T cells were maintained in Dulbecco’s modified Eagle medium (Lonza) supplemented with 10% of FBS (Sigma), 100 U/ml penicillin, and 100 μg/ml streptomycin (Thermo Fisher Scientific).

### Lentiviral vectors

All lentiviral vectors used in this paper are derivatives of TM952 (57), which is a Prom_CMV_-MCS-IRES-neo construct. TM1116 and TM1119 are derivatives of TM952 that code for 3xHA-huRSK1 or RSK4, respectively. These vectors were constructed using the Gateway technology (Invitrogen) from donor plasmids Hs.RPS6KA1, Hs.RPS6KA6 kindly provided by Dominic Esposito through the Addgene collection (Addgene refs: 70573 and 70579, respectively). BLP32 is a TM952 derivative coding for As-RSK1 (hu-RSK1 with analog-sensitive kinase mutation: Leu141 into Ala). BLP34 is a TM952 derivative coding for As-RSK4 (hu-RSK4 with analog-sensitive kinase mutation: Leu152 into Ala).

Lentiviruses were produced in HEK293T cells grown in a well of a 6-well plate by cotransfection of 0.75 μg of pMD2-VSV-G (VSV-glycoprotein), 1.125 μg of pMDLg/pRRE (Gag-Pol), 0.625 μg of pRSV-Rev (Rev), and 2.5 μg of lentiviral vector, using the transfection regent TransIT-LT1 (Mirus Bio). Supernatants containing the lentivirus were collected 48 h post transfection and filtered through a 0.45 μm filter. For transduction, 10,000 cells/well (24-well plate) were infected with 2 times 100 μl of lentivirus.

### DNA constructs

The original plasmid encoding human TRIM33 (pCS2-FLAG-hEcto/Tif1g) was obtained from Dr Stefano Piccolo through Addgene (#20902). This DNA construct was used as template for generating HA-tagged TRIM33 in the pcDNA3 backbone. The S1119A mutant was generated using the QuikChange methodology (Stratagene).

### GST-S6 recombinant production, purification, and *in vitro* kinase assay

Protocol was as in (18) but using plasmids pTM1116, pTM1119, pBLP32, and pBLP34 (corresponding to RSK1-WT, RSK4-WT, As-RSK1, and As-RSK4 respectively).

### Western blotting

Cells were directly collected in Laemmli buffer. Samples were heated at 100 °C for 5 min and run on 8 to 10% Tris-glycine SDS polyacrylamide gels and then transferred to PVDF (for HA-RSK detection or some Thiophosphate ester blots) or nitrocellulose membranes. Membranes were blocked for 1 h at room temperature with TBS-5% milk (Regilait). Primary antibodies were diluted in blocking solution and incubated overnight at 4 °C. Antibodies used are as follows: anti-HA (mouse, MMS101P – Covance, 1/4000), anti-RxxS∗/T∗ (phospho-RSK substrates, rabbit, CST9614 – Cell signaling technology, 1/1000), anti-thiophosphate ester (rabbit, NBP2-67738 – Novusbio, 1/1000, anti-ß actin (mouse, A5441 – Sigma, 1/10,000), anti-RanBP3 (rabbit, CST93706 – Cell signaling technology, 1/1000), anti-PDCD4 (rabbit, CST9535 – Cell signaling technology, 1/1000), anti-ZC3H11A (mouse, H00009877B01P – Abnova, 1/1000), anti-IRS2 (rabbit, CST3089 – Cell signaling technology, 1/1000), anti-SORBS2 (mouse, SAB4200183 – Sigma-Aldrich, 1/1000), anti-RSK1/2/3 (rabbit, CST9347 – Cell signaling technology, 1/1000), anti-p-Akt (Ser473; rabbit, CST9271 – Cell signaling technology, 1/1000), anti-Tubulin (mouse, T5168 – Sigma, 1/1000), anti-p-RSK (Ser380; rabbit, CST9341 – Cell signaling technology, 1/1000), anti-HA (mouse, clone 12CA5, ROAHA – Sigma, 1/1000). Membranes were washed for 15 min with TBS-0,1% Tween 20 (3 washes). Secondary antibodies were diluted in blocking solution and incubated for 1 h at room temperature. Secondary antibodies used are as follows: anti-rabbit or anti-mouse coupled to HRP (Dako, 1/5000) and anti-rabbit IgG light chain coupled to HRP (Millipore, 1/5000). Membranes were then washed three times, as previously, and once with TBS. Detection was performed with SuperSignal West chemiluminescence substrate (Pico or Dura, Thermo Fisher scientific) or Westar Supernova (Cyanagen). Images were captured by conventional films (Fig. 5) or with cooled CCD cameras (Fusion Solo S - Vilber or Odyssey FC - Li-Cor) (Figure 1, Figure 2, Figure 3, Figure 4, Figure 5 and S2).

### Thiophosphorylation in permeabilized cells

One million five hundred thousand HeLa RSK1-WT, As-RSK1, RSK4-WT, or As-RSK4 cells were seeded in 10-cm dishes. For the screen of thiophosphorylated peptides enrichment, three dishes were used per condition, for the validation of candidates *via* immunoprecipitation; ½ dish was used per immunoprecipitation. The next day, cells were treated with PMA (200 nM) for 15 min at 37 °C 5% CO2, in order to activate RSKs. Following this step, cells were permeabilized with 500 μl of analog-kinase buffer + PMA (20 mM Hepes pH 7.5, 100 mM KOAc, 5 mM NaOAc, 2 mM MgOAc_2_, 1 mM EGTA, 20 μg/ml digitonin, 10 mM MgCl_2_, 0.5 mM DTT, 1× phosphatase inhibitor cocktail 2 (P5726, Merck), 1× cOmplete protease inhibitor (11697498001, Roche), 57 μg/ml creatine kinase (Calbiochem 238,395), 5 mM creatine phosphate (Calbiochem, 2380), 0.1 mM ATP, 0.1 mM N6-Bn-A∗TP analog (BioLog), 3 mM GTP (Roth, K056.4) and 200 nM PMA), in order for the ATP analog to enter the cells and thus allow the thiophosphorylation reaction to happen. Reaction proceeded for 1 h at 37 °C, 5% CO_2_ on a rocking plate.

### Thiophosphorylated peptides enrichment

Cells were scraped and collected (∼1.5 ml) into a 2 ml tube and sonicated. Forty microliters of lysate were collected and alkylated with PNBM (2.5 mM) for 2 h at room temperature. Alkylation reaction was then stopped by the addition of 20 μl Laemmli 3× buffer (= these samples are the lysates samples to control proper thiophosphorylation in the experiment). The rest of the lysate (not alkylated) was probe-sonicated three times for 15 s. Clear lysates were obtained by centrifugation at 15,000*g* for 10 min. Protein concentration was determined by Bradford protein assay according to the manufacturer’s instructions (Bio-Rad #5000006). Samples (2000 μg) were incubated 1 h at 55 °C and 1500 rpm with 10 mM tris(2-carboxyethyl) phosphine hydrochloride (Thermo Fisher Scientific #77720) and then precipitated by methanol/chloroform in Eppendorf 15 ml LoBind tubes. One volume sample was mixed with four volumes of −20 °C methanol and one volume of −20 °C chloroform and incubated 20 min on ice. Three volumes of cold water were added and thoroughly mixed and incubated 5 min on ice before being centrifugated at 5000*g* for 15 min at 4 °C. The upper phase was removed and proteins at the interphase were precipitated by the addition of eight volumes of −20 °C methanol and centrifugation at 5000*g* for 5 min at 4 °C. Pellets were air-dried and resuspended in 500 μl of 50 mM ammonium bicarbonate and transferred to 1.5 ml Eppendorf LoBind tube. Trypsin (Promega #V5111) was added at a 1:50 ratio μg trypsin: μg lysate. Samples were incubated overnight at 37 °C and 850 rpm. An additional 20 μg trypsin was added 2 h prior to quenching enzymatic activity by adjusting to pH 2 with TFA. Samples were centrifugated at 15,000*g* 10 min and the supernatants were transferred in a new Eppendorf 1.5 ml LoBind tube. Samples were concentrated to 40 μl in a Speedvac. After the concentration, one sample volume of 200 mM Hepes pH7.3 and two sample volume of 100% acetonitrile were added to samples. The pH was adjusted to 7.0 with hydrochloric acid 1 M. Per sample, 100 μl of Sulfolink Coupling Resin (Thermo Fisher Scientific #20401) was washed once with 1 ml of Hepes 200 mM. All the wash steps of Sulfolink Coupling Resin were done by centrifugation of the resin 1 min 2000*g* at room temperature and by aspiration of the supernatant using a Pasteur pipette attached to a vacuum hose. Prior to sample addition to the resin, 5 μl of 5 μg/ml bovine serum albumin was added. Samples were rotated overnight with Sulfolink Coupling Resin at room temperature in the dark. Samples were washed once with 1 ml of each of the following solutions: ddH_2_O, 5 M sodium chloride, 50% acetonitrile, 5% formic acid. The samples were incubated with 1 ml of 10 mM DTT for 10 min with rotation at room temperature. After centrifugation and aspiration of the DTT, 100 μl 1 mg/ml oxone (Sigma #228036) was added to the resin and recovered after centrifugation. Following this first elution, 200 μl 1 mg/ml oxone was added to samples for 25 min with agitation at room temperature 850 rpm and recovered and pooled to the first elution by centrifugation. A last elution was done and pooled with the previous with 50 μl 1 mg/ml oxone. Per sample, one SDB-RPS stageTips (Affinisep #TiPS-RPS.T1.200.96) was conditioned first with 20 μl methanol then with 40 μl loading/wash1 buffer (TFA 1%, Isopropanol 99%) taking care that the stageTips does not dry up. The 350 μl of oxone elution was mixed with 100 μl loading/wash1 buffer and loaded on SDB-RPS StageTips and centrifuged. All the centrifugation step for StageTips were done at 1500*g* for 5 min. Wash StageTips once with 100 μl loading/wash1 buffer then once with wash2 buffer (TFA 0.2%, acetonitrile 5%). StageTips were transferred to collection tube and two elution were done with each time 30 μl fresh elution buffer (20 μl of NH_4_OH added to 4 ml of 60% (vol/vol) ACN). The tubes were placed immediately in a speedvac at 45 °C to dryness, then reconstituted in 8 μl of solvent A (0.1% TFA in 2% acetonitrile). Peptides were directly loaded onto reversed-phase precolumn (Acclaim PepMap 100, Thermo Fisher Scientific) and eluted in backflush mode. Peptide separation was performed using a reversed-phase analytical column (Acclaim PepMap RSLC, 0.075 × 250 mm, Thermo Fisher Scientific) with a linear gradient of 4 to 27.5% solvent B (0.1% formic acid in 80% acetonitrile) for 100 min, 27.5%-40% solvent B for 10 min, 40 to 95% solvent B for 1 min, and holding at 95% for the last 10 min at a constant flow rate of 300 nl/min on an Ultimate 3000 RSLC system.

### Mass spectrometry

Peptides were analyzed by an Orbitrap Fusion Lumos tribrid mass spectrometer (Thermo Fisher Scientific). The peptides were subjected to NSI source followed by tandem mass spectrometry (MS/MS) coupled online to the nano-LC. Intact peptides were detected in the Orbitrap at a resolution of 120,000. Peptides were selected for MS/MS using HCD setting at 30; ion fragments were detected in the Orbitrap at a resolution of 60,000. A data-dependent procedure that alternated between one MS scan followed by MS/MS scans was applied for 3 s for ions above a threshold ion count of 5.0E4 in the MS survey scan with 30.0 s dynamic exclusion. The electrospray voltage applied was 2.1 kV. MS1 spectra were obtained with an AGC target of 4E5 ions and a maximum injection time of 50 ms, and MS2 spectra were acquired with an AGC target of 1E5 ions and a maximum injection set to 110 ms. For MS scans, the m/z scan range was 325 to 1800. The resulting MS/MS data were processed using Sequest HT search engine within Proteome Discoverer 2.5 SP1 against a Human database protein database obtained from Uniprot. Trypsin was specified as a cleavage enzyme allowing up to two missed cleavages, four modifications per peptide, and up to five charges. A mass error was set to 10 ppm for precursor ions and 0.1 Da for fragment ions. Oxidation on Met (+15.995 Da), phosphorylation on Ser, Thr, and Tyr (+79.966 Da), conversion of Gln (−17.027 Da) or Glu (−18.011 Da) to pyro-Glu at the peptide N-term were considered as variable modifications. False discovery rate was assessed using Percolator and thresholds for protein, peptide, and modification site were specified at 1%. For abundance comparison, abundance ratios were calculated by comparing the number of detected PSMs and by label-free quantification of the precursor intensities within Proteome Discoverer 2.5 SP1.

### Immunoprecipitation after thiophosphorylation

Cells were lysed by adding 500 μl of lysis buffer 2× (100 mM Tris–HCl pH 8, 200 mM NaCl, 1% NP40, 4 mM EDTA and one tablet of phosphatase/protease inhibitor (Thermo Fisher Scientific) per 5 ml of lysis buffer) to the 500 μl of analog-kinase buffer. Cell lysis happened for 15 min at 4 °C, then lysates were homogenized by 10 passages through 21G needles and cleared by centrifugation at 12,000*g* for 10 min at 4 °C. Supernatants were transferred to a new tube where the pre-clearing step was performed with the addition of 30 μl (per dish) of protein A/G magnetic beads (Pierce) and incubation at 4 °C for 30 min. Pre-cleared lysates were then collected. A 200 μl sample of this lysate (per condition) was transferred to a new tube containing 100 μl of Laemmli 3× (=cell lysate control). The rest of the lysate was incubated with 4 μg of antibody for 2 h at 4 °C. Antibodies used are as follows: anti-RanBP3 (rabbit, CST93706 – Cell signaling technology), anti-PDCD4 (rabbit, CST9535 – Cell signaling technology), anti-ZC3H11A (mouse, H00009877B01P – Abnova), anti-IRS2 (rabbit, CST3089 – Cell signaling technology), anti-SORBS2 (mouse, SAB4200183 – Sigma-Aldrich), and anti-HA (mouse, clone 12CA5, ROAHA – Sigma). A/G beads (30 μl/condition) were added to the lysate and incubated 2 h at 4 °C. A/G beads were then washed 3× with lysis buffer 1× for 5 min at 4 °C. Beads were finally resuspended in 40 μl of kinase buffer 1× (25 mM Hepes pH 7.5, 50 mM NaCl, 20 mM ß-glycerophosphate, 1 mM DTT, 20 mM MgCl_2_, 100 μM Na_3_VO_4_) and alkylated with PNBM (2.5 mM) for 2 h at room temperature. 3× Laemmli buffer was added to stop the alkylation reaction and IP proteins were separated from the beads after heating at 100 °C for 5 min.

### Transfection and treatment of cells with RSK activators/inhibitors

HEK293 cells were transfected by calcium-phosphate precipitation as previously described (58). Cells were grown for 24 h after transfection and serum-starved overnight using serum-free Dulbecco’s modiffied Eagle’s medium where indicated. Starved cells were pretreated with PD184352 (10 μM) or BI-D1870 (10 μM) (Biomol), where indicated, and stimulated with PMA (50 ng/ml), EGF (25 ng/ml), insulin (100 nM), or FBS (FBS; 10%) before being harvested. Unless indicated otherwise, all drugs and growth factors were purchased from Invitrogen.

### Statistical analysis on phospho-peptides enrichment

Label-free quantification was performed by obtaining the area under the curve for each peptide using Proteome Discoverer 2.5; no normalization was applied. The quantitative values for the different replicates were first combined, log-transformed, and median-normalized using the QFeatures Bioconductor package (59). In order to cope with the numerous drop-outs in peptide intensities and the peptide presence/absence patterns in particular, the proDA method (27) was applied. The linear model included one indicator variable representing replicated experiments and a second one representing the experimental groups of interests (AS/WT and RSK1/RSK4). Then, AS *versus* WT effects for each peptide were tested for RSK1 and RSK4, using two-sided Wald tests. All *p*-values were adjusted using Benjamini–Hochberg corrections (60).

### Gene ontology analysis

Protein overrepresentation in biological processes was analyzed using the PANTHER test (Released 20231017) with the homo sapiens GO database (https://doi.org/10.5281/zenodo.7942786) and the Fisher's Exact test.

## Data availability

The mass spectrometry proteomics data have been deposited to the ProteomeXchange Consortium *via* the PRIDE partner repository (Perez-Riverol *et al.*, 2022, Nucleic Acids Res 50: D543-D552) with the dataset identifier PXD047020 and 10.6019/PXD047020.

## Supporting information

This article contains supporting information.

## Conflict of interest

The authors declare that they have no conflicts of interest with the contents of this article.
