# Supplementary material for: Identification of RSK substrates using an analog-sensitive kinase approach
Source: J Biol Chem. 2024 Feb 10;300(3):105739. doi: 10.1016/j.jbc.2024.105739 (PMC10945272; doi:10.1016/j.jbc.2024.105739)
Supplement: Supporting Figures S1 and S2 [file mmc1.docx]

**Identification of RSK substrates using an analog-sensitive kinase approach.**

Belén Lizcano-Perret^1^, Didier Vertommen^2^, Gaëtan Herinckx^2^, Viviane Calabrese^3^, Laurent Gatto^4^, Philippe P. Roux^3,5^ & Thomas Michiels^1^*

**Supporting information**

**
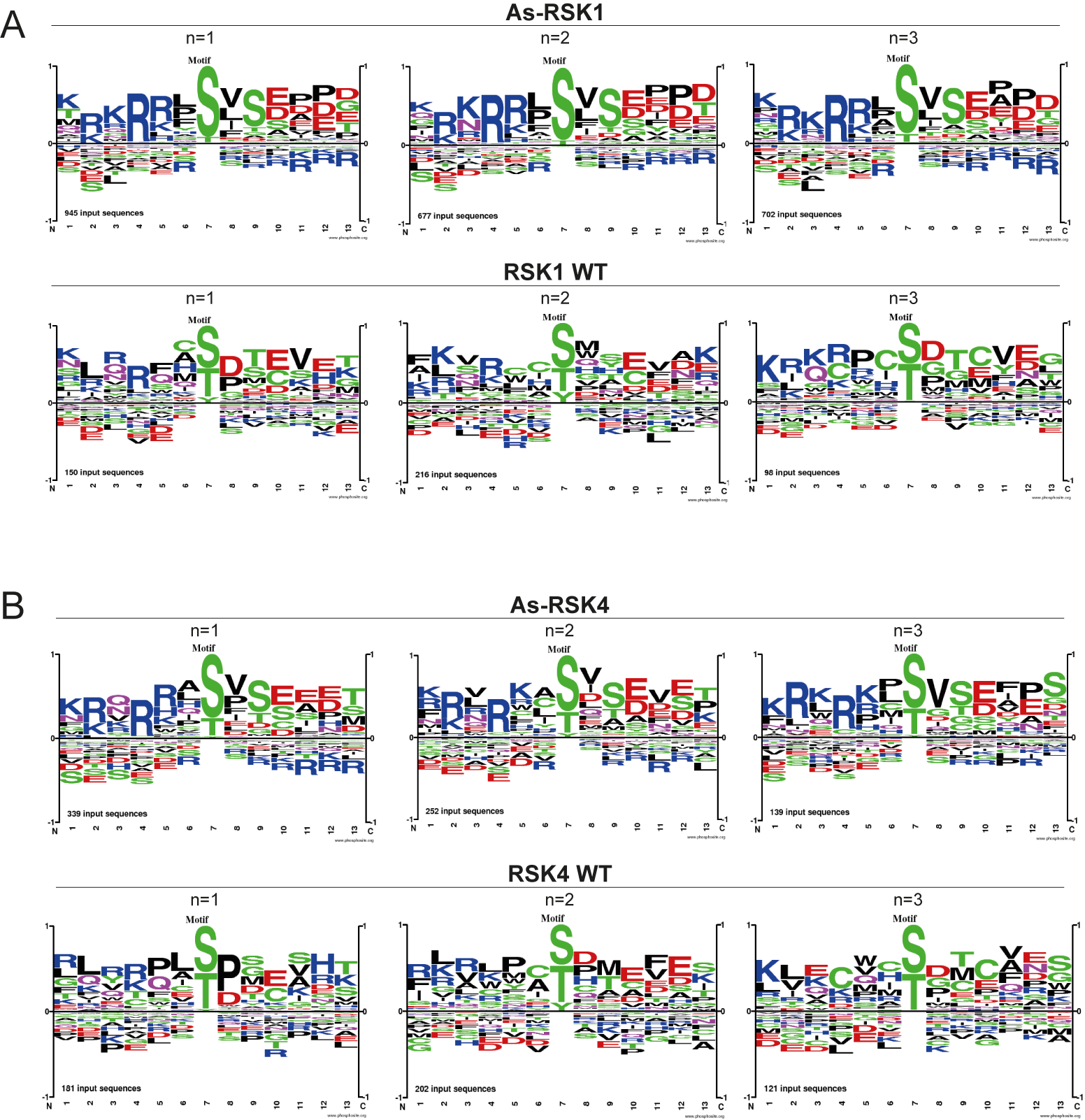
**

**Figure S1. Phosphorylation motifs of phosphopeptides thiophosphorylated by (A) As/WT-RSK1 or (B) As/WT-RSK4.** All phosphorylated peptides identified in each triplicate were used to create the posphorylation motif. Phosphorylation motifs were made with: phosphosite.org


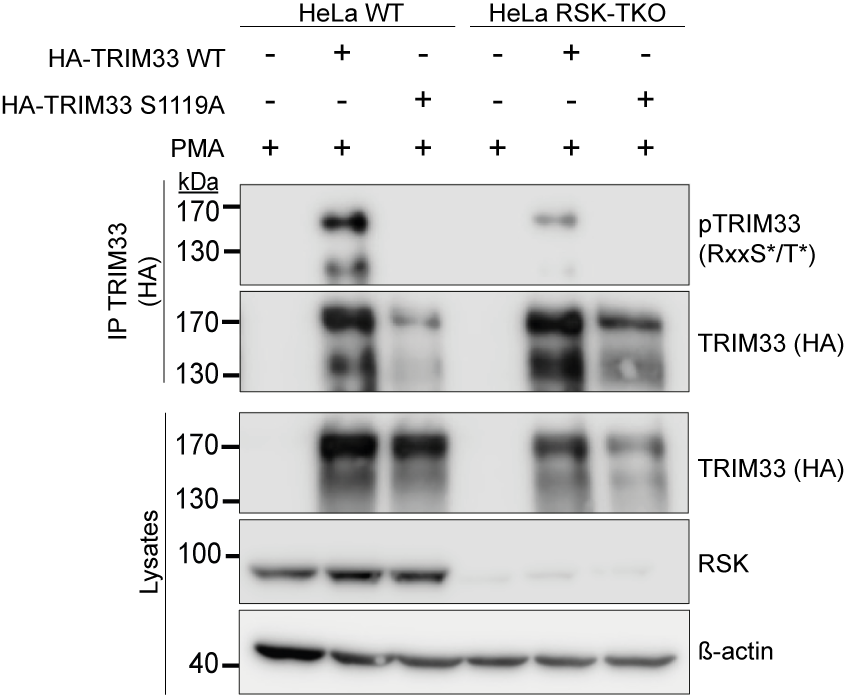


**Figure S2. TRIM33 phosphorylation in RSK TKO cells.** HeLa WT or RSK1-2-3 triple-KO cells were transfected with HA-tagged TRIM33 and treated for 30 min with PMA before TRIM33 (HA) immunoprecipitation. Phosphorylation of TRIM33 was assessed by western blot using the phospho-RSK substrates (phospho-RxxS*/T*) antibody.
